# Understanding and forecasting phreatic eruptions driven by magmatic degassing

**DOI:** 10.1186/s40623-018-0855-z

**Published:** 2018-05-18

**Authors:** John Stix, J. Maarten de Moor

**Affiliations:** 1 Department of Earth and Planetary Sciences, McGill University, 3450 University Street, Montreal, QC H3A 0E8 Canada; 2 Observatorio Vulcanológico y Sismológico de Costa Rica (OVSICORI), Universidad Nacional, AP 2386-3000, Heredia, Costa Rica

**Keywords:** Phreatic eruptions, Magmatic inputs, Overpressure, Sealing, Vaporization, Forecasting

## Abstract

This paper examines phreatic eruptions which are driven by inputs of magma and magmatic gas. We synthesize data from several significant phreatic systems, including two in Costa Rica (Turrialba and Poás) which are currently highly active and hazardous. We define two endmember types of phreatic eruptions, the first (type 1) in which a deeper hydrothermal system fed by magmatic gases is sealed and produces overpressure sufficient to drive explosive eruptions, and the second (type 2) where magmatic gases are supplied via open-vent degassing to a near-surface hydrothermal system, vaporizing liquid water which drives the phreatic eruptions. The surficial source of type 2 eruptions is characteristic, while the source depth of type 1 eruptions is commonly greater. Hence, type 1 eruptions tend to be more energetic than type 2 eruptions. The first type of eruption we term “phreato-vulcanian”, and the second we term “phreato-surtseyan”. Some systems (e.g., Ruapehu, Poás) can produce both type 1 and type 2 eruptions, and all systems can undergo sealing at various timescales. We examine a number of precursory signals which appear to be important in understanding and forecasting phreatic eruptions; these include very long period events, banded tremor, and gas ratios, in particular H_2_S/SO_2_ and CO_2_/SO_2_. We propose that if these datasets are carefully integrated during a monitoring program, it may be possible to accurately forecast phreatic eruptions.
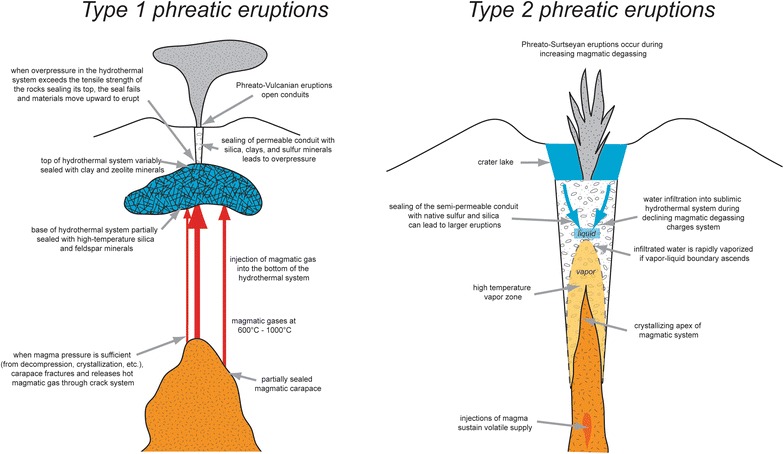

## Introduction

Phreatic eruptions are sudden events, commonly with few if any precursors. They can be lethal to people close by, and they commonly precede larger magmatic eruptions. Phreatic eruptions result from rapid heating and vaporization of fluids which are commonly situated at shallow levels beneath a volcano. By definition, there is no solid juvenile material in the eruption products (although sometimes this can be difficult to determine). The fluids involved in phreatic eruptions may originate by downward percolation of meteoric fluids into hot rocks or a hot conduit. They also may form from upward migration of volcanic fluids, including gases, supercritical fluids, and melts, into a hydrothermal system or shallow aquifer. A combination of the two scenarios is also possible.

A critically important question during phreatic activity is determining if magma is involved or not. In other words, what is the severity of the crisis? Is a phreatic eruption simply a one-off event with little or no magmatic contribution? Or does it involve significant amounts of magmatic gas and potentially eruptible magma? Commonly the answer to this question is ambiguous. Barberi et al. (1992) have pointed out that most large eruptions are preceded by phreatic activity, so it is crucial to ascertain at an early stage if magma is involved or not. Hence, this paper specifically addresses magmatic inputs, the mechanisms involved, and the signs and signals indicative of a magmatic signature.

The lack of solid juvenile material in eruptive products is not necessarily an indication that magma is absent at shallow levels. This is a very difficult issue, as Cashman and Hoblitt (2004) retrospectively (i.e., > 20 years later) recognized juvenile glass in some deposits erupted from Mt. St. Helens in spring 1980 prior to the climactic eruption on 18 May. Hence these precursory eruptions were phreatomagmatic, not phreatic in nature. On the other hand, Pardo et al. (2014) indicate that caution is warranted if fresh-looking glassy material is discovered, as there may be few differences between lithic and juvenile glassy material in many cases.

The purpose of this paper is to focus on magmatic contributions (gas, supercritical fluids, and/or melt) to phreatic eruptions. We first examine six systems where significant insight has been obtained on phreatic activity. Then, we present a conceptual model of phreatic activity associated with shallow magma. The model encompasses two types of phreatic eruptions: (1) type 1, in which magmatic contributions into an overlying and variably sealed hydrothermal system result in overpressure and eventually phreato-vulcanian eruptions; (2) type 2, whereby the magmatic inputs vaporize confined near-surface liquid water, causing overpressure and eventually phreato-surtseyan eruptions. We end by discussing the manner by which phreatic activity can be forecast, also assessing the means by which magmatic contributions can be recognized.

### Terminology and definitions

Phreatic eruptions are a broad class of volcanic eruptions, perhaps most easily defined in contrast to purely magmatic eruptions. Magmatic eruptions are driven by processes occurring as magmas rise through the crust whereby exsolution of volatiles and crystallization lead to overpressure and eruption. Magmatic eruptions are classified into subcategories according to the style and character of magma ejection. The classification scheme of magmatic eruptions is historically derived from key locations or events that displayed distinct eruptive behavior, i.e., hawaiian, strombolian, vulcanian, and plinian.

Phreatic eruptions are eruptions in which magmatic processes are not the principal driving mechanism. Rather, broadly defined hydrothermal processes (i.e., interactions among water, rocks, and magmatic heat and gas) play the key role in generating phreatic eruptions. On our wet planet, explosive interactions between volcanoes and meteoric water are extremely diverse. Phreatic eruptions encompass steam-driven explosions generated by magma intruding fluvial sediments and aquifers, lava or pyroclastic flows interacting with surface water, geyser-like explosions driven by depressurization of near boiling-point subterranean geothermal water, and volcanic eruptions expelling hydrothermal systems formed during periods of repose (e.g., Barberi et al. 1992; Rouwet et al. 2014). In contrast to magmatic eruptions, a broadly accepted classification scheme of phreatic eruptions is lacking.

This paper specifically focuses on phreatic eruptions at volcanic hydrothermal systems that are fed by magmatic input. We consider two defining characteristics of these phreatic eruptions to be (1) the dominance of hydrothermally altered or lithic components in the eruptive products and (2) the involvement of exogenous water (i.e., steam/water not immediately exsolved from melt). The lack of juvenile material in eruptive products is a clear indicator that magma was not directly involved in eruption, yet a minor component of fresh glassy material may be accidental and does not necessary preclude an eruption from being phreatic in nature (e.g., Christenson et al. 2010; Pardo et al. 2014). Vaporization and expansion of exogenous water is a fundamental process driving phreatic eruptions (e.g., Rouwet et al. 2014). Exogenous water includes surface water such as lakes, rivers, and seawater, meteoric aquifers such as those found in basin fill conglomerates, geothermal fluids found in mature volcano-hosted hydrothermal systems, and acidic brines found in immature magmatic–hydrothermal systems. In the latter systems, water derived from condensation of magmatic gas may be a significant or even dominant source of liquid water (e.g., Giggenbach 1992). Thus, exogenous water can include water that is principally derived from magmatic fluids but has experienced hydrothermal processing such as phase changes (e.g., condensation) and reaction with host rock.

Phreatomagmatic eruptions display characteristics of magmatic eruptions and phreatic eruptions. Phreatomagmatic eruptions are driven by magmatic processes but also involve vaporization of exogenous water as a contributing force (Zimanowski et al. 2015). Deposits from phreatomagmatic eruptions have a clear component of juvenile material that displays textural evidence for quenching and fragmentation in response to violent interaction with coolant (e.g., Zimanowski et al. 2015; Alvarado et al. 2016). Eruptive products can be dominated by juvenile material or altered/lithic material. Eruptions in which hydrothermal processes are dominant in initiating the eruption, but which entrain passive or residual magma that does not play a significant role in driving the eruption, can still be considered phreatic (e.g., Christenson et al. 2010).

## Six significant phreatic systems

We now examine six systems which have experienced phreatic eruptions. These examples have been chosen because (1) the datasets are good and (2) both type 1 and type 2 phreatic eruptions are illustrated. For example, Ruapehu and Poás usually host acid crater lakes. As a result, type 2 eruptions are commonly generated at these volcanoes from extensive vaporization of liquid water caused by magmatic input. At times, however, both systems undergo sealing and pressurization, sometimes resulting in type 1 eruptions and demonstrating that near-surface hydrothermal systems can be subject to these processes, as well as their deeper brethren. Information on these six systems is summarized in Table 1.

**Table 1:** Summary of signals and changes prior to phreatic eruptions

Volcano and dates	Observed signal (s)	Elapsed time between first appearance of signal and eruption	Date of eruption	Eruption type	Eruption forecast?	Inferred processes	Sources of data
Nevado del Ruiz 1985	Banded tremor	7 days	11-Sep-85	1	No	Injection of magmatic gas into the hydrothermal system	Martinelli (1990)
	Tremor	3 days	13-Nov-85	1?	No	Magmatic eruption	Voight (1990)
Aso 1994–1995	VLP events	350–400 s	Various	1	Possibly	Injection of magmatic gas into the hydrothermal system	Kaneshima et al. (1996)
Ontake 2007	Inflation + VT swarms	~ 3 months	Mid-late March 2007	1	No	Magma intrusion followed by injection of magmatic gas into the hydrothermal system	Nakamichi et al. (2009)
	VLP event	~ 2 months					
	LP signals, tremor	~ 1–1.5 months					
Ontake 2014	VT seismicity	~ 1 month	27-Sep-14	1	No	Injection of magmatic gas into the hydrothermal system	Terakawa et al. (2016)
	LP seismicity	~ 20 days					Kato et al. (2015)
	VLP event	25 s					Maeda et al. (2015a)
	He isotopes	Months to years					Sano et al. (2015)
Ruapehu 2007	Decrease in lake temperature	~ 7 months	25-Sep-07	1	No	Injection of magmatic gas into the hydrothermal system	Christenson et al. (2010)
	Decrease in SO_2_ flux	~ 3 months					
	Increase in CO_2_/SO_2_	~ 5 months					
	Negative lunar-seismic correlation	~ 3 months					Girona et al. (2018)
Turrialba 2014	LP seismicity	~ 2 weeks	29-Oct-14	1	Possibly	Injection of magmatic gas into the hydrothermal system	de Moor et al. (2016a)
	VT seismicity						de Moor et al. (2017b)
	Increase in CO_2_/SO_2_						
	Decrease in SO_2_ flux						
Poás 2014, 2017	Decrease in CO_2_/ SO_2_	Days	2014 (60 eruptions)	2	No	Pulses of magmatic gas into the shallow hydrothermal system	de Moor et al. (2016b)
	Increase in SO_2_ flux	1–2 weeks	2017	1	Possibly	Destruction of hydrothermal seal	de Moor et al. (2017c)
	VT seismicity	~ 2 weeks					
	LP seismicity	~ 2 weeks					
White Island 2012	Increased spring emissions	~ 2 years	2012–2013	2	No	Increased magmatic degassing	Christenson et al. (2017)
	Increase in SO_2_ flux						
	Decrease in CO_2_/SO_2_						
	Deflation						
	Increased RSAM						
Rincón de la Vieja 2014, 2017	Decrease in CO_2_/SO_2_	Months?	Sept–Oct 2014	2	No	Increased magmatic degassing	de Moor et al. (2016b)
	Decrease in H_2_S/SO_2_	Weeks	Feb–March 2017	2	No	Increased magmatic degassing	Battaglia et al. (2017)

### Nevado del Ruiz 1985 (Colombia) (type 1 phreatic eruption)

**Fig. 1:**
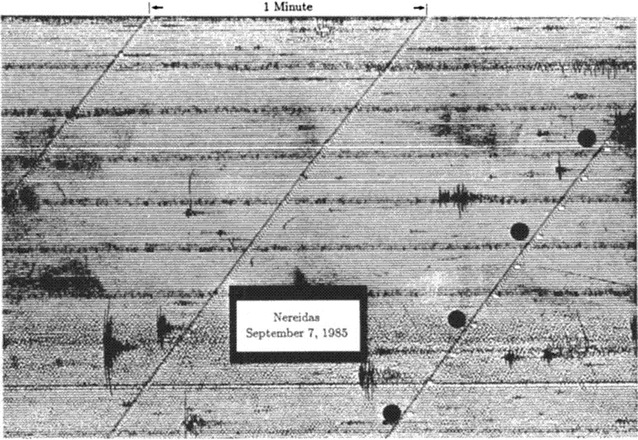
Banded tremor observed on 7 September 1985 at the Nereidas seismic station located ~ 4 km south of the summit of Nevado del Ruiz. This tremor was measured 4 days prior to a phreatic eruption on 11 September. Each tremor cycle is 15–20 min’ duration. Reprinted from the Journal of Volcanology and Geothermal Research, Volume 41, Martinelli, B., “Analysis of seismic patterns observed at Nevado del Ruiz volcano, Colombia during August–September 1985”, pages 297–314, Copyright 1990, with permission from Elsevier

Nevado del Ruiz is a large andesitic stratovolcano near the northern end of the Northern Volcanic Zone of the Andes. After about a year of precursory unrest, Nevado del Ruiz erupted explosively on 13 November 1985, melting the summit ice cap and generating lahars which killed ~ 25,000 people (Hall 1990). A smaller eruption 2 months earlier on 11 September was termed phreatic at the time, based on the lack of juvenile material emitted by the eruption (Hall 1990; Voight 1990). Distinctive seismic signals, termed “banded tremor”, were observed to begin several days before this phreatic eruption (Martinelli 1990, 1991). On 5 September tremor bands were first noted. The bands were typically 15–20 min long, while individual tremor cycles (tremor signal + quiescent interval) had durations of ~ 90–100 min (Martinelli 1990) (Fig. 1). Individual tremor bands had mean dominant frequencies of ~ 4 Hz and showed a gradual amplitude increase followed by a sudden cessation (Martinelli 1990).

Giggenbach et al. (1990) proposed a model for the magmatic–hydrothermal system beneath the volcano whereby continued crystallization of old magma releases heat and gas to the overlying hydrothermal system. This hydrothermal system is situated at comparatively shallow levels (probably less than 1 km deep), allowing mixing to occur between magmatic fluids from below and meteoric fluids percolating downward from above. The banded tremor studied by Martinelli (1990) may represent periodic injections of hot, magmatically derived gas into the hydrothermal system, causing boiling to occur, hence pressurization and subsequent eruption. Giggenbach et al. (1990) hypothesized that the eruptions were ultimately driven by release of hot gas from crystallizing magma (i.e., second boiling). A similar process at shorter timescales was proposed for Galeras volcano in Colombia (Stix et al. 1997).

### Aso 1994–1995 (Japan) (type 1 phreatic eruptions)

Aso is a large Quaternary caldera on the island of Kyushu. Within the caldera is situated a smaller andesite volcano, which is active. Kaneshima et al. (1996) studied this system for a year in 1994–1995 during which a series of phreatic eruptions occurred. During larger phreatic events, they observed a remarkable sequence of characteristic seismic events. Prior to an eruption, a very long period displacement (VLPD) was observed for 350–400 s, with an initial accelerating inflationary phase followed by an approximately linear deflationary phase. The durations of inflation and deflation were roughly equal, and the eruption was closely associated with the peak of inflation. Superimposed on the inflation was a series of long period pulses (LPP) with a dominant period of 15–20 s. The LPP signals increased significantly in magnitude during inflation, then rapidly dissipated after the inflation peak. Short period tremor (SPT) with a dominant frequency of 0.3–0.5 s began just prior to the inflation peak, then strengthened significantly as deflation was initiated. The SPT signals ended with the cessation of deflation.

Kaneshima et al. (1996) interpret the VLPD inflation as a gradual increase in fluid pressure due to injection of heat and gas from magma at deeper levels. In this model, the LPP signals are the direct manifestation of this injection of magmatic materials into the hydrothermal system situated at shallow levels beneath the crater. Hence, both the VLPD and LPP seismicity record the increasing pressurization of the hydrothermal system as hot magmatic gases vaporize hydrothermal fluids, causing them to boil. At a critical overpressure exceeding the tensile strength of the impermeable cap rocks sealing the top of the hydrothermal system, the rocks fail and fluids discharge and erupt by opening a conduit to the surface crater, generating the SPT seismicity and allowing the hydrothermal reservoir to deflate. No large LPP signals are observed during deflation because the system is depressurizing.

### Mount Ontake 2007 and 2014 (Japan) (type 1 phreatic eruptions)

Mount Ontake is a stratovolcano which has been the site of numerous phreatic eruptions in both prehistoric and historic time. In the past several decades, phreatic eruptions have occurred in 1979, 1991, 2007, and 2014 (Yamaoka et al. 2016). The 2014 eruption on 27 September was fatal to 58 people with five more missing. The 2007 eruption was associated with a temporally interesting sequence of events. Shallow magma intrusion occurred in December 2006–January 2007 as indicated by inflation and volcanotectonic (VT) earthquake swarms (Nakamichi et al. 2009). The intrusion occurred at the same time as an increase in ^3^He/^4^He from 7.0 to 7.2 *R*_A_ in gases from Nigorigo located 4.2 km from the summit (Sano et al. 2015). Based on seismic and geodetic measurements, the shallow magma was intruded into the volcano at depths as shallow as ~ 3 km, releasing heat and gas and pressurizing the hydrothermal system above (Nakamichi et al. 2009). A very long period (VLP) signal was recorded on 25 January 2007 and interpreted by Nakamichi et al. (2009) as groundwater vaporization (boiling causing inflation) followed by the opening of a crack and deflation. The crack likely did not reach the surface, as no eruption followed. Hence, this may have been a failed eruption. Long period (LP) signals and tremor were observed in February–March 2007, indicating that an elevated level of pressurization was maintained within the shallow hydrothermal system. The phreatic eruption took place in mid-late March (the exact date is unknown). Afterward, LP events were rare, although some tremor persisted. The complete sequence from December to March can be seen as progressive pressurization of the magmatic–hydrothermal system driven by shallow intrusion of magma.

No inflation was recorded prior to the 2014 eruption (Yamaoka et al. 2016). VT seismicity was first observed in late August 2014 about a month before the eruption, with intensification beginning on 6 September, while ~ 27 LP events were noted between 8 September and the eruption on 27 September (Kato et al. 2015). Terakawa et al. (2016) suggest that the system was pressurized for at least several weeks prior to the eruption causing stress changes, with local VT seismicity dominated by normal faulting before the eruption compared to dominantly reverse faulting afterward. A VLP event was recorded 25 s before the start of the eruption (Maeda et al. 2015a). SO_2_ fluxes of more than 2000 tons/day were recorded immediately after the eruption, with 450 tons/day measured on 9 October and 130–140 tons/day on 20–21 November (Mori et al. 2016). Together, the lack of deformation, the VT earthquake swarms, the predominantly normal VT faulting, and the elevated SO_2_ fluxes suggest that a release of high-temperature gas from shallow magma occurred in late August 2014, initiating the chain of events which pressurized the shallow hydrothermal system and led to the fatal eruption 1 month later. The source of the gas is enigmatic. The magmatic gas may have been generated by continued cooling and crystallization of the magma since 2007 (second boiling). The gas could also be the product of new magma emplaced at shallow levels since 2007, although there is no clear evidence for this. Yamaoka et al. (2016) propose that the hydrothermal source was located at 1–2 km depth. If so, this suggests that the hydrothermal system may have shallowed over time since the 2007 eruption when it was located at 2–3 km depth (Nakamichi et al. 2009).

### Ruapehu 2007 (New Zealand) (alternating type 1 and 2 phreatic eruptions)

Ruapehu is an active stratovolcano which undergoes periodic eruptions through a summit crater lake that exhibits wide variations in temperature depending upon the activity of the volcano. Type 2 phreatic and phreatomagmatic eruptions commonly occur from extensive vaporization of liquid water caused by high heat flow through the system (Christenson and Wood 1993; Sherburn et al. 1999). The eruption of 25 September 2007 was different; it ejected mainly lithic debris, but up to 5% of the material emitted consisted of juvenile glass. Strictly speaking this was a phreatomagmatic eruption, but the system’s behavior showed many aspects of a type 1 phreatic eruption, in particular its highly sealed nature prior to the eruption (Christenson et al. 2010). During the year leading up to the eruption, the lake temperature declined from 25–30 °C to < 15 °C, a condition indicative of efficient vent blockage and sealing beneath, preventing input of magmatic heat to the surface lake system. At the same time, CO_2_ fluxes were both highly variable (70–660 tons/day) and generally declining, with a comparatively low flux of 180 tons/day 1 month before the eruption. SO_2_ fluxes increased from near zero during January–October 2016 to 34–55 tons/day in March–June 2007 before declining to 13 tons/day on 23 August 2007 1 month prior to the eruption. Molar CO_2_/SO_2_ ratios decreased from values near 60 in late 2006 to a value of ~ 5 in April–May 2007 before increasing to ~ 20 soon before the eruption. The magmatic CO_2_/SO_2_ ratios of 5 and measurable SO_2_ emissions are indicative of input of magmatic gases into the system, although Christenson et al. (2010) suggest that the sulfur could have been remobilized from accumulations within the hydrothermal system which had previously sealed it. The decreasing lake temperatures, increasing CO_2_/SO_2_ ratios, and low CO_2_ and SO_2_ fluxes in August 2007 immediately prior to the eruption clearly indicate a well-sealed system (Christenson et al. 2010). Girona et al. (2018) have demonstrated a significant, negative lunar-seismic correlation at this time caused by low permeabilities (~ 10^−10^ m^2^), consistent with the sealing hypothesis. After the eruption, lake temperatures climbed to 32–38 °C in late 2007 and early 2008. At times CO_2_ and SO_2_ fluxes, respectively, exceeded 2000 and 500 tons/day, while the CO_2_/SO_2_ ratio was maintained at a level near 5 indicating magmatic conditions. In summary, the 25 September 2007 eruption altered the hydrothermal system beneath the crater lake from one that was nearly fully sealed beforehand to one afterward that was nearly fully open, allowing essentially pure magmatic gases to flow to the surface (Christenson et al. 2010).

Several minutes prior to the eruption and during the eruption itself, a series of VLP, VT, and tremor signals were recorded. Two pre-eruptive VLP events appear to have been sourced at 3–7 km depth, accompanied by VT events sourced at ~ 4 km and shallow tremor at 1–2 km. According to Jolly et al. (2010), it is unclear whether the VLP signals are the result of fluid pressurization, depressurization of the system by the VT events, or a combination of both. A third VLP signal during the eruption, which was accompanied by tremor, was significantly shallower at ~ 1.5 km below the crater lake (Jolly et al. 2010).

### Turrialba 2010–2017 (Costa Rica) (type 1 phreatic eruptions)

**Fig. 2:**
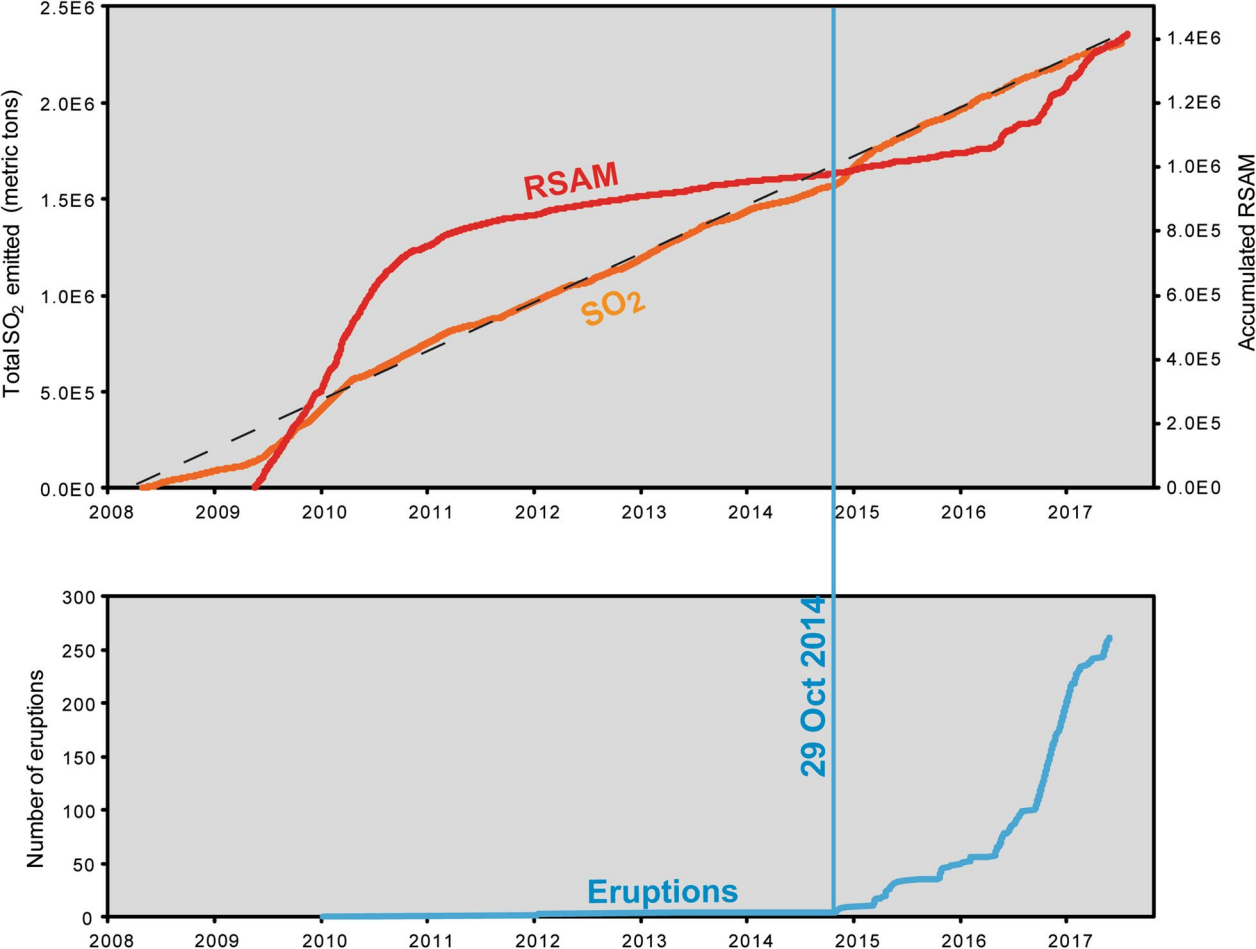
Turrialba monitoring data for 2008–2017 showing cumulative real-time seismic amplitude measurement (RSAM), SO_2_ emissions, and eruptions during this period. Note the episodic RSAM behavior, the steady output of SO_2_, and the significant increase in eruptive activity beginning in 2015. Periods of low SO_2_ flux before the 29 October 2014 eruption suggest partial sealing of the system beforehand. [Reproduced with permission from Conde et al. (2014) and de Moor et al. (2016a, 2017b)]

Unrest at Turrialba volcano began in the mid 1990’s with distinct increases in seismicity and degassing after ~ 150 years of dormancy. A change in fumarole chemistry showed a clear indication of reactivation with the appearance of SO_2_ in low temperature fumaroles in November 2001 (Vaselli et al. 2010). Thereafter, SO_2_ fluxes increased from ~ 1 ton/day in 2002 to ~ 740 tons/day in 2008 (Martini et al. 2010) and peaked in mid-2009 at ~ 3500 tons/day (Conde et al. 2014). Seismicity at Turrialba is best described as highly diverse, with volcanotectonic, long period, tornillo, and very long period events, as well as gliding and harmonic tremor, all observed (Martini et al. 2010). Seismicity (RSAM) peaked at Turrialba in 2009–2010, and the first phreatic eruption occurred on 5 January 2010, which opened a jetting fumarole vent. Thereafter, RSAM and SO_2_ flux both decreased to more moderate levels, although remaining well above background levels for ~ 1.5 years (Fig. 2). Another phreatic eruption occurred in January 2012, which opened a second jetting fumarole vent. The vents simultaneously emitted ash in May 2013. A new period of increased activity began on 29 October 2014 with an energetic blast and sustained ash eruptions thereafter, and another strong blast occurred on 9 December 2014. Distinctive bombs of silicified polymict breccia were erupted during these events, and were interpreted as components of the sealed conduct (de Moor et al. 2016a). This activity was accompanied by increased RSAM and SO_2_ flux, which continued during the following months of quiescence in terms of ash emissions (de Moor et al. 2016a). A new period of eruptive activity occurred in March–April 2015, with less energetic eruptions but sustained ash emissions that had significant societal impact, blanketing the capital city of San José with ash and disrupting flights. Low-energy ash emissions occurred in October 2015, and sporadic semi-continuous periods of ash emissions have been occurring through 2016 and 2017.

The activity at Turrialba can be described as a slow re-awakening process, with vent-opening eruptions becoming generally more frequent with time (Fig. 2). Eruptive products were initially characterized as phreatic in character, but a small proportion of fresh glassy material was later recognized (Alvarado et al. 2016). However, the composition of this allegedly juvenile material was very similar to the composition of scoria from the 1855–1856 magmatic eruption, thus calling into question whether this material was truly juvenile or remobilized from the previous magmatic system/products (either shallow quenched dikes, near-surface vesiculated material, or residual shallow magma; de Moor et al. 2016a). The “juvenile” component from the 2014–2015 eruptions displays quench features consistent with interaction with liquid water, as well as subtle chemical alteration of clast surfaces (Alvarado et al. 2016). Bombs from the 29 October 2014 eruption included blocks of silicified hydrothermal breccia, providing evidence that the system was hydrothermally sealed, or at least partially, since SO_2_ fluxes were still significant in the months beforehand (de Moor et al. 2016a). Later eruptive products from 2015 to 2017 contained true juvenile material (Alvarado et al. 2016; Rizzo et al. 2016), and spatter bombs deformed at impact were observed in the crater (de Moor et al. 2017b). However, the initial ash samples highlight the intrinsic difficulty of determining whether eruptions should be termed “phreatic” or “phreatomagmatic”.

**Fig. 3:**
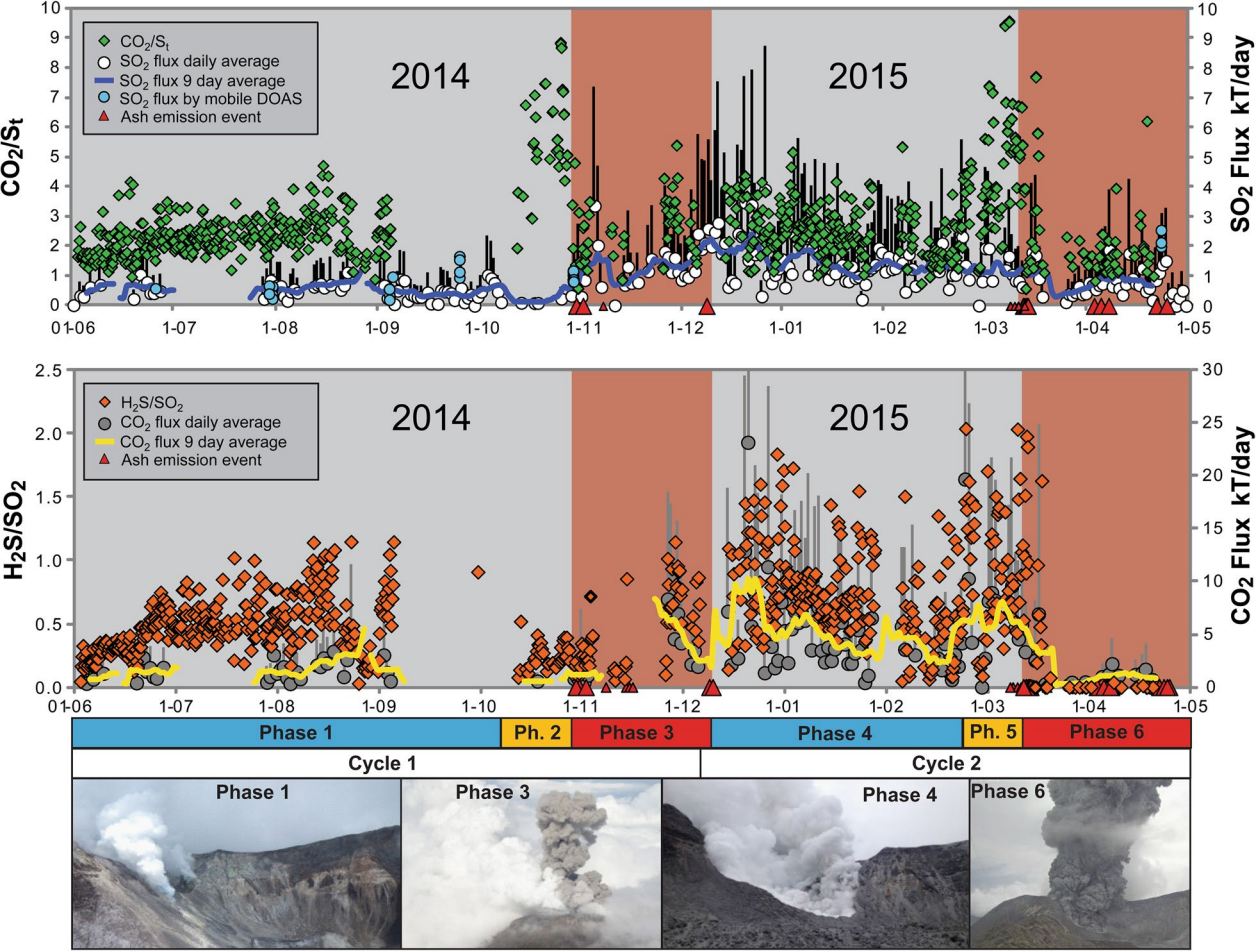
High-resolution gas monitoring at Turrialba in 2014 and 2015. Note the increases in CO_2_/S_T_ prior to ash emission events. Note water-rich gas plumes in phases 1–3 (photos). The disappearance of H_2_S in early March 2015 marks the rapid transition from hydrothermally influenced to purely magmatic degassing, interpreted to reflect expulsion of the hydrothermal system and conduit opening. Reproduced with permission from de Moor et al. (2016a) and used courtesy of the American Geophysical Union

The eruptions at Turrialba are a prime example of the type of phreatic or phreatomagmatic eruptions that accompany volcano activation after a prolonged period of dormancy. The slow ramping up of the activity at Turrialba allows detailed study of the eruptions. High-frequency gas monitoring during this period highlights two key aspects of the activity (Fig. 3): (1) the occurrence of magma intrusion events identified through high CO_2_/SO_2_ precursors to the eruptive activity, and (2) expulsion of the hydrothermal system and opening of magmatic conduits identified through the disappearance of H_2_S (de Moor et al. 2016a). Thus, Turrialba-type “phreatic” eruptions are accurately described as vent-opening eruptions in response to new magma injections that disrupt and eventually expel the hydrothermal system.

### Poás 2006–2017 (Costa Rica) (alternating type 1 and 2 phreatic eruptions)

Phreatic eruptions at Poás are distinct in character from the vent-opening eruptions at Turrialba. Phreatic eruptions occur through a hyper-acid crater lake. Thus, interaction with external water is obvious in contrast to eruptions at Turrialba. Phreatic bursts were very common during 2006–2016, with hundreds recorded seismically per year. Many smaller “gas bursts” (eruption columns less than 10 m above the lake surface) also occurred but were not detectable seismically. Eruptions are characterized by a “cockscomb” ejection of lake water, lithics, sediment ± minor juvenile material that is erupted from the lake to heights ranging from 1 m to more than 500 m, accompanied by dilute lateral base surges of steam. In April 2017, a more explosive phreatomagmatic eruption changed the system dramatically and the lake was completely vaporized, revealing two high-flux jetting fumarolic vents that presumably were the sources of the eruptions in the period 2012–2016.

The variations in magmatic gas flux to the lake appear to drive the systematic short-term variations in CO_2_/SO_2_ observed at Poás (de Moor et al. 2016b). Essentially, sulfur chemistry drives these variations. At low temperature and higher pH (low gas flux), sulfur scrubbing reactions result in molar CO_2_/SO_2_ ratios typically greater than ~ 0.7. At high temperature and low pH (high gas flux), the scrubbing reactions in the lake are less efficient, resulting in lower CO_2_/SO_2_ below 0.4; these values are similar to ratios observed in the high-temperature magmatic gases at the Poás dome fumaroles. Thus, when gas input to the lake decreases, the CO_2_/SO_2_ ratio increases and phreatic eruptions occur (de Moor et al. 2016b). Long-term variations at Poás follow similar principles, but CO_2_/SO_2_ varies to much larger degrees over several orders of magnitude.

### Synthesis of observations

In the cases of Nevado del Ruiz and Aso, the geophysical data support a model of magmatic fluid injection into a shallow hydrothermal system. At Ruiz, banded tremor was observed for 1 week before the 11 September 1985 eruption. At Aso, VLPD and LPP events were recorded. These signals imply pressurization of the hydrothermal system. At Ontake, magma intrusion occurred in late 2006 and early 2007 followed by a phreatic eruption, while in 2014 an accumulation of magmatic gas led to another phreatic eruption. Turrialba is notable both for its extended awakening process and its evolution from “hydrothermal” behavior to “magmatic” behavior, as shown by the gas data (de Moor et al. 2016a). Monitoring of CO_2_/SO_2_ provided clear deep precursors to eruptions, whereas monitoring of H_2_S/SO_2_ ratios was particularly insightful in terms of understanding the shallower processes involved. At Turrialba, it was also difficult during the initial eruptions (2014–2015) to distinguish between juvenile and lithic glass (de Moor et al. 2016a; Alvarado et al. 2016).

Both Ruapehu and Poás produce type 2 phreatic eruptions at times of high heat flow from vaporization of liquid water. Yet both systems are capable of generating type 1 eruptions. At Ruapehu, there are multiple lines of evidence suggesting that the shallow hydrothermal system was well sealed prior to the 25 September 2007 eruption (Christenson et al. 2010), with the eruption breaking the seal and forming a direct conduit to the surface for magmatic gas release. At Poás, a similar progression was associated with the April 2017 eruptions (de Moor et al. 2017c).

Sealing and unsealing is common and occurs repeatedly at various timescales in phreatic systems. Sealing can be a progressive process occurring over several years, but it also may occur rapidly, sometimes at timescales as short as days or weeks. A key challenge is to identify the sealing process and its rate of formation in real time, unambiguously distinguishing this process from a transition into repose.

## A model for magmatically driven phreatic eruptions

### Magmatic–hydrothermal interactions

It is clear that many if not most phreatic eruptions are the result of interaction between magma emplaced at comparatively shallow crustal levels (0–5 km) and an aquifer or hydrothermal system above the magma. The hydrothermal system thus acts as a buffer, receiving high-temperature magmatic input (gas, supercritical fluids, melt) at its base, and releasing fluids and rock at its top through a conduit system which reaches the surface (see de Moor et al. 2016a; Giggenbach et al. 1990; Kaneshima et al. 1996).

For these types of systems, there are three common elements: magmatic gas input, hydrothermal sealing, and vaporization of liquid water. Hydrothermal sealing provides the means by which a hydrothermal system can pressurize. Sealing can occur in all hydrothermal systems ranging from deep (i.e., several km) to near-surface. The sealing process appears to occur over a range of timescales from fast (e.g., days) to slow (e.g., months to years), and this element of time is clearly significant in terms of when an eruption occurs. If one can identify when and why the sealing process is occurring, this may be a means to identify a system which is undergoing pressurization and may erupt. Finally, all hydrothermal systems will vaporize when magmatic fluids are injected into them. For deeper hydrothermal systems, the process of vaporization will cause overpressure and may be manifested by geophysical signals such as banded tremor, LP, and VLP events. For shallow or near-surface systems including those with crater lakes, the vaporization process will be extensive and the volcano’s response potentially rapid in terms of phreatic activity.

Both wet and dry volcanoes have the potential to produce phreatic eruptions. The abundance of surface and near-surface water on wet volcanoes results in a large volume expansion as liquid water is vaporized by magma and/or magmatic gas. This mechanism drives phreatic eruptions. For comparatively dry volcanoes hosting a hydrothermal system, the injection of magma and/or magmatic gas into the hydrothermal envelope has two consequences. First, magmatic water condenses; second, liquid hydrothermal water is vaporized. Both processes occur at high temperatures; under such conditions, host rocks undergo alteration while sulfur precipitates as magmatic gas condenses (Christenson et al. 2010). The end result is the development of a seal. Such conditions are conducive to phreatic eruptions. A key question is the sealing rate, i.e., how fast can a seal develop?

The ability of the hydrothermal system to trap and absorb magmatic heat and gas depends on four main factors. First, the size of the hydrothermal system in terms of its areal extent and thickness is important. Small, thin aquifers cannot take up significant magmatic inputs; the magmatic gases should be able to efficiently pass through the aquifer to the surface, while also drying the aquifer in the process. By contrast, a well-developed hydrothermal system as seen at Nevado del Ruiz should have significantly greater buffering capacity. Second, the depth of the hydrothermal system should play a fundamental role in the energy and explosivity of phreatic eruptions, as shallow hydrothermal seals should fail more easily under less overpressure. Third, the size of the magmatic system will play a significant role. A single, small-volume intrusion of magma will have significantly less impact than either a larger volume of magma or a series of magma intrusions. Related to this is the depth at which the intruding magma stops moving upward. A magma that stalls at 5 km depth should have less dramatic effects than one which reaches 1 km depth, in which case the effects will be much more strongly felt. For example, differences in depth may be the reason why some eruptions emit juvenile material (shallow magma), while others do not (deeper magma). Fourth, whether the magma is dynamic or static is an important element. A magma that is ascending, decompressing, and releasing gas will generate significant effects that vary in space and time as the rising magma approaches the hydrothermal interface. This was likely the case at Turrialba in 2009–2015. Conversely, a magma which is static will be cooling, crystallizing, and releasing gas in some sort of steady-state fashion. The overlying impacts and effects should be likewise steady-state in large part. Such a scenario may occur at Aso and Ontake.

We now present two conceptual models for type 1 and type 2 phreatic systems. A key common element for both types is that they receive hot magmatic fluids (gases) from deeper levels. Key differences are that type 1 systems are commonly deeper and seal themselves, allowing pressurization to develop, while type 2 systems appear to be more open and shallow in nature. Type 1 systems typically exhibit vulcanian eruptive activity, while type 2 systems are associated with surtseyan activity. However, we note that some systems, notably Ruapehu and Poás, can exhibit both type 1 and type 2 behavior at various times.

### Type 1 systems

**Fig. 4:**
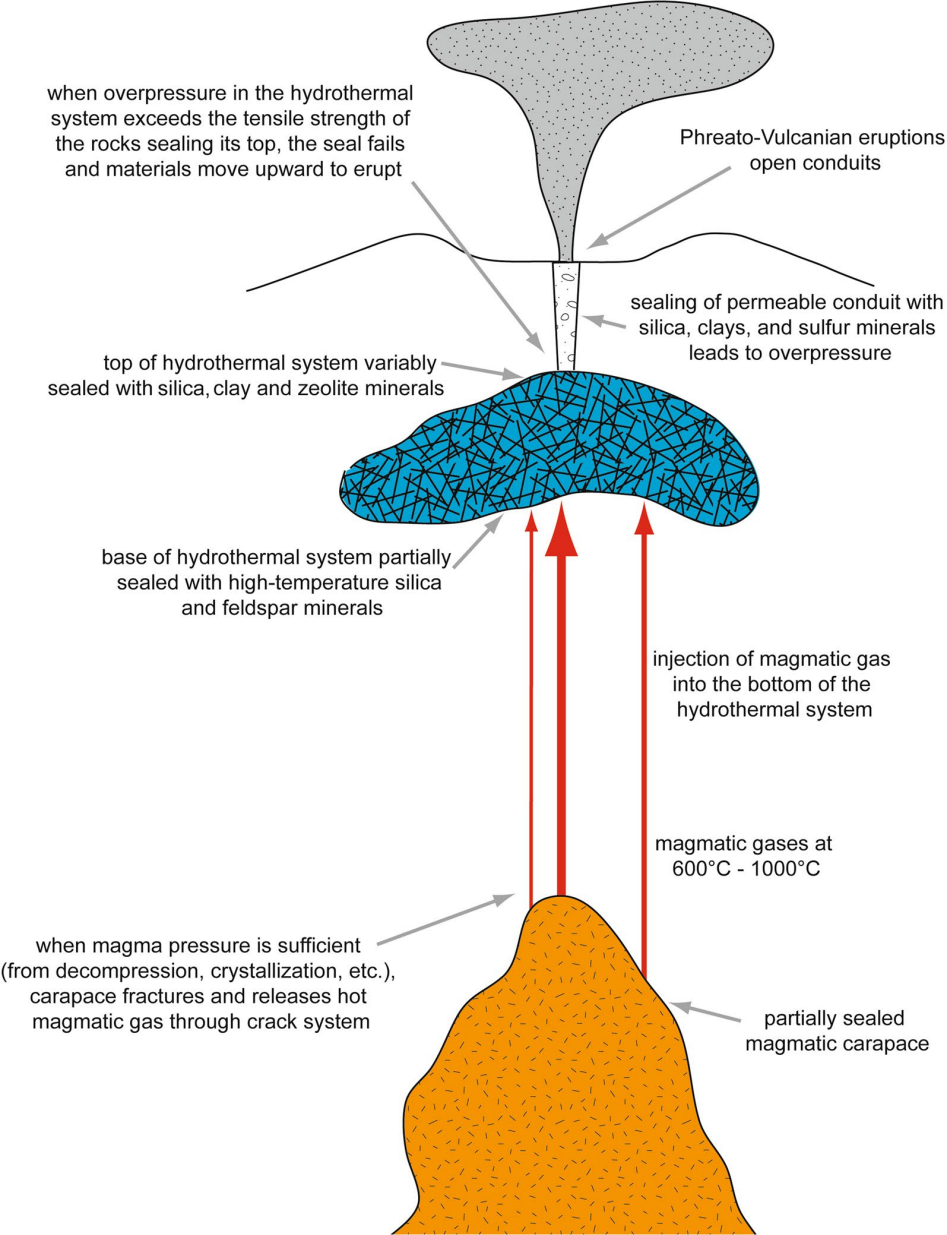
Model for type 1 phreatic systems and eruptions. A shallow magma body releases gas by intrusion and/or crystallization. The gases are transported upward through a series of cracks, intersecting the hydrothermal system above. If the hydrothermal system is sealed at its top, the system will become pressurized from the addition of hot magmatic gases. Such conditions promote phreatic eruptions

Figure 4 shows a schematic view of a type 1 phreatic system. The hydrothermal system is underlain by a magma body which periodically releases gases upward into the hydrothermal system. The magmatic gases are released by intrusion, crystallization, or a combination of the two processes. A magma body enclosed by a solidified carapace provides a partial seal which ruptures and releases gas when magmatic overpressure exceeds the tensile strength of the carapace. If the carapace is absent or poorly developed, magmatic gases can escape continually. The hydrothermal system also may be sealed at its base and/or top. The bottom seal can rupture when magmatic gases arrive. If the upper seal is strong, overpressure will build in the hydrothermal system. A deep hydrothermal system can generate more overpressure than a shallow system due to the greater lithostatic overburden on the system. The degree of alteration also plays a role in determining the strength and extent of the seal. At a critical level of overpressure, the upper seal will rupture, allowing gases and lithic debris to be transported through a conduit system and erupted at the surface. If the overpressure does not reach this critical level, an eruption will not occur, although there may be seismic and gas signals (e.g., long period events, elevated H_2_S/SO_2_, low gas fluxes) indicating that the system is sealed and overpressured. Hazards associated with these eruptions are ejection of ballistics to significant distances (> 1 km; e.g., Ontake), small pyroclastic flows and surges, and emission of fine ash that can travel significant distance with impacts on air traffic (e.g., Turrialba).

### Type 2 systems

**Fig. 5:**
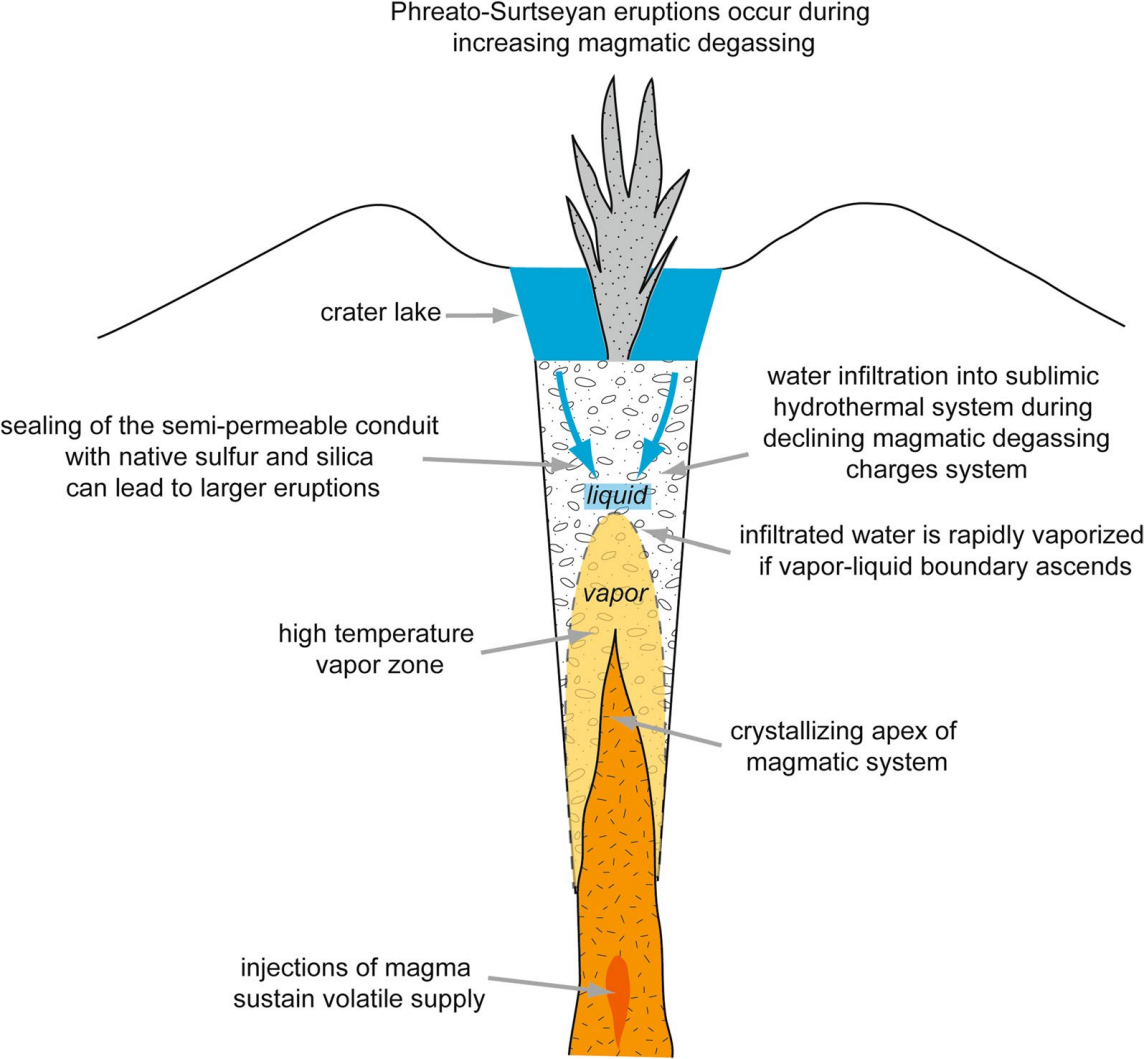
Model for type 2 phreatic eruptions at Poás. Increasing magmatic gas input into the lake raises the vapor–liquid boundary, resulting in vaporization of confined liquid water, generating volume change, pressurization, and eruption. Compared to type 1 phreatic systems, here the conduit is more open with a shallower magma system. Type 2 phreatic eruptions are also very common at Rincon de la Vieja and other hyperacid crater lake systems. The eruptive style is more akin to surtseyan eruptions than vulcanian eruptions which are more related to type 1 phreatic activity

The hyperacid crater lake at Poás has emitted significant amounts of SO_2_ and other magmatic gases (de Moor et al. 2016b). Such behavior has been recognized at other crater lake systems as well (Shinohara et al. 2015; Tamburello et al. 2015). De Moor et al. (2016b) showed that gases emitted from Poás crater lake vary systematically, with a decrease in the CO_2_/SO_2_ ratio preceding phreatic eruptions. They present a model in which transient variations in magmatic gas flux (i.e., power supply) to the lake result in decreasing CO_2_/SO_2_ prior to eruptions and also drive phreatic eruptions (Fig. 5). By this model, phreatic eruptions occur during periods when the magmatic input to the lake is actively increasing, and do not occur when the input is decreasing. The key consideration is whether the vapor–liquid boundary in the sublimnic hydrothermal system is rising or falling. If the boundary is rising, confined liquid water is vaporized, driving phreatic eruptions. If the boundary is falling, infiltration of hydrothermal fluids into the sublimnic zone loads the system for the next eruption. Many volcanoes hosting shallow hydrothermal systems with abundant surface water may display these types of phreatic eruptions, including Rincón de la Vieja (Costa Rica), Kawah Ijen (Indonesia), White Island and Ruapehu (New Zealand), and Copahue (Argentina). Hazards associated with these types of eruptions include ejection of crater lakes and generation of lahars (e.g., Kawah Ijen) and wet pyroclastic surges (e.g., Ruapehu). Ballistics and ash emissions typically have less impact compared to type 1 eruptions due to the very wet nature of type 2 eruptions, which tends to restrict the aerial extent of the finer eruptive products.

## Forecasting phreatic eruptions

To our knowledge, no phreatic eruption has been formally and accurately forecast as such or in terms of its size and timing. However, the technical tools and understanding of phreatic eruptions driven by magmatic gas should allow quantitative assessment that can inform hazard assessment. We envision volcanoes prone to phreatic eruptions being monitored using multiple parameters (geophysical and geochemical) feeding into automated probabilistic calculations forecasting the likelihood of eruption within a given time period. Here, we examine parameters by which to forecast phreatic eruptive activity. Because these eruptions are commonly small and sudden events, precursory signals may be subtle or absent (Maeda et al. 2015b). In our opinion, the application of broadband seismicity and gas ratios offer the most useful and cost-effective means for forecasting. If these two types of data can be integrated on a real-time or near real-time basis, new insight may be gained in terms of our ability to forecast phreatic eruptions. Other techniques such as deformation may also be helpful, although many phreatic eruptions appear to occur with no significant precursory inflation, which would suggest no pressure buildup and a type 2 eruption mechanism. However, the lack of recognized deformation may be a result of the shallow deformation source in the hydrothermal system or upper conduit. Monitoring of systems prone to phreatic eruptions, which do not necessarily involve significant magma movement, will be more likely to detect subtle changes that do occur prior to explosions if instruments are deployed closer to the conduit than would normally be the case for volcanoes prone to magmatic eruptions.

### Very long period seismicity

The very long period signals discussed above appear to have common behavior in terms of an inflation–deflation mechanism. The inflationary phase is ascribed to pressurization, commonly from injection of hot magmatic gas into a cooler hydrothermal reservoir system causing boiling. The deflation results from evacuation of the reservoir as the seal ruptures from excessive overpressure, allowing fluid discharge upward through a propagating crack system.

At Aso VLP signals were of long duration (350–400 s) and shallowly sourced (1–1.5 km deep) (Kaneshima et al. 1996). At Ontake in 2007, a VLP event located at ~ 2.4 km was observed 2 months before the eruption (Nakamichi et al. 2009). By contrast, in 2014, a VLP event was recorded only 25 s before the eruption and was shallowly sourced (< 1 km) (Maeda et al. 2015a). At Ruapehu a series of two VLP events, VT earthquakes, and tremor were observed several minutes prior to eruption. The VLP events were thought to originate at deep levels (3–7 km) (Jolly et al. 2010).

Mayon volcano in the Philippines periodically experiences phreatic eruptions without clear precursors (Catane and Mirabueno 2001; Maeda et al. 2015b). During a phreatic eruption on 7 May 2013, a VLP event was recognized and interpreted as a subhorizontal tensile crack slightly offset from the crater at a depth of ~ 240 m (Maeda et al. 2015b). These authors suggest that the lack of clear precursors could be due to progressive sealing of the shallow hydrothermal system.

While these data indicate that VLP events can originate at different depths, their occurrence also appears to be an indication of pressurization–depressurization sequences leading to phreatic eruptions. Their recognition is thus an important tool in assessing the probability of a future eruption, although predicting such eruptive events cannot clearly be done using the VLP data alone.

### Banded tremor

Banded tremor is an unusual seismic signal characterized by periods of tremor interspersed with periods of quiescence. The duration of both the tremor and the quiescent periods are sometimes constant, producing a striking pattern on a seismogram, as can be seen in Fig. 1 (Martinelli 1990, 1991). The occurrence of banded tremor commonly precedes explosive eruptive activity (Martinelli 1990; Gresta et al. 1996; McKee et al. 1981). Hence, it is an important precursory signal.

The banded tremor described at Nevado del Ruiz was clearly significant in terms of short-term forecasting. For the 11 September 1985 phreatic eruption, banded tremor was first recorded on 5 September 1 week beforehand (Martinelli 1990). For the devastating 13 November magmatic eruption 2 months later, Voight (1990) reported that 3 days of continuous tremor commenced on 10 November before the eruption, although it is unclear if this tremor was banded. Very similar banded seismic signals were observed at Mt. Etna, Italy, in March–May 1987 associated with two phreatic eruptions on 8 April and 17 April (Gresta et al. 1996). Banded tremor was first recorded on 1 April, 1 week before the first eruption. Individual tremor bands lasted 25–35 min and quiescent spacings between bands 140–160 min, for total cycle durations of 165–195 min. During this 1-week interval, tremor amplitudes increased progressively to the time of eruption, then disappeared temporarily before resuming on 13 April, 4 days before the second eruption.

In contrast to these short-term eruption indicators, banded tremor at Karkar volcano, Papua New Guinea, was significantly longer-lived during unrest in 1978–1979. McKee et al. (1981) suggest that a small body of magma intruded to shallow levels in 1977–1978, as manifested by elevated seismicity, incandescence to 1000 °C, and widespread fumarolic activity. Banded tremor began in July 1978, 6 months prior to the initiation of phreatic explosive activity in January 1979. The tremor strengthened appreciably in late August and was associated with increased gas emissions from the craters. Tremor amplitudes peaked in late October and declined thereafter. In late January 1979, the color of the gas emissions changed from white and blue to dense white vapor. A second important decline in tremor amplitude occurred in early February, while LP events began to be recorded in mid-February at rates of 15–40 events/day. Taken together, these changes in early 1979 may signal sealing and/or pressurization of the shallow hydrothermal system. The most significant eruption, which was phreatic in nature containing no juvenile material, occurred on 8 March 1979.

The Nevado del Ruiz and Mt. Etna examples illustrate that banded tremor can serve as a short-term precursor to phreatic explosive activity. The Karkar activity demonstrates that occurrence of banded tremor can also extend over an appreciable time period. Nevertheless, the tremor at Karkar was clearly associated with explosive activity which itself extended over 8 months (January–August 1979). For this type of phreatic activity observed at these and other volcanoes, the presence of magma at shallow levels appears to exert a significant influence on the overlying hydrothermal or groundwater system.

### Gas emissions

Gas emission monitoring has high potential as an eruption forecasting tool for phreatic eruptions. This is based on the fact that hydrothermal and magmatic gases are vastly different in character; thus, small changes in a volcano’s hydrothermal system result in significant and readily detectable changes in gas composition (de Moor et al. 2016a). The delivery of heat from magmatic systems to overlying hydrothermal systems essentially occurs through the upward migration of high-temperature magmatic fluids. Thus, the fundamental process responsible for driving phreatic eruptions should also be quantifiable and measurable through the gases emitted. However, the lack of gas emission can be equally important, as this could signify the formation of a hydrothermal seal, resulting in accumulation of gas and pressure in the hydrothermal system and ultimately leading to phreatic eruptions (e.g., Christenson et al. 2010). Distinguishing between a transition to quiescence and a transition to sealing with ongoing gas input is a key issue requiring integrated assessment of multiple monitoring parameters such as deformation and seismicity. Additionally, sealing could conceivably result in pressurization without eruption, potentially shutting off magmatic input from below.

The field of volcanic gas monitoring is experiencing rapid technological advances. New methods for in situ and remote measurements of gas flux and gas composition have recently been developed (e.g., Aiuppa et al. 2005; Shinohara 2005; McGonigle et al. 2008; Kern et al. 2015). Hydrothermal–magmatic systems that typically produce phreatic eruptions are rather challenging for monitoring when exclusively using gas fluxes (e.g., SO_2_ fluxes; Galle et al. 2010), as the emissions from these systems tend to be low compared to more magmatic systems. This is primarily due to the fact that systems with high gas and heat fluxes cannot easily establish and maintain hydrothermal systems because meteoric water is rapidly boiled off (Pasternack and Varekamp 1997). By contrast, volcanoes with inherently lower magmatic gas output can form extensive hydrothermal systems, which interact with magmatic volatiles introduced from below. These reactions remove reactive volatiles from the magmatic gas phase (Symonds et al. 2001). Sulfur dioxide is the primary gas species used for remote monitoring of gas flux, and is also a reactive gas, forming sulfuric acid via a process known as “scrubbing”. Two fundamental dissociation reactions dominate this process (e.g., Kusakabe et al. 2000):

1. $$ 4{\text{SO}}_{{ 2 ( {\text{g)}}}} + \, 4{\text{H}}_{2} {\text{O}}_{{ ( {\text{l)}}}} \to {\text{H}}_{2} {\text{S}}_{{ ( {\text{g)}}}} + \, 3{\text{H}}_{2} {\text{SO}}_{{ 4 ( {\text{aq)}}}} $$
2. $$ 3{\text{SO}}_{{ 2 ( {\text{g,aq)}}}} + \, 2{\text{H}}_{2} {\text{O}}_{{ ( {\text{l)}}}} = {\text{ S}}_{{ ( {\text{s,l)}}}}^{\text{o}} + \, 2{\text{HSO}}_{{ 4 ( {\text{aq)}}}}^{ - } + \, 2{\text{H}}^{ + }_{{ ( {\text{aq)}}}} $$

Reaction 1 produces H_2_S, another gas useful for monitoring, and sulfuric acid. Reaction 2 does not produce a gas species, but instead native sulfur and sulfuric acid. Deeper hydrothermal systems associated with more explosive phreatic eruptions tend to be associated with H_2_S (e.g., Turrialba and Ontake; de Moor et al. 2016a; Mori et al. 2016), whereas shallower hydrothermal systems fed by air-saturated water (more oxidizing conditions) generate eruptions which can be H_2_S-poor (e.g., Poás; de Moor et al. 2016b). The sulfur chemistry at these latter systems is probably dominated by reaction 2, but both reactions can be active at the same time, or may vary in relative significance with time, space, and volcanic activity.

Carbon dioxide is another very important gas species in these systems because it is abundant and readily measurable, and behaves very differently to sulfur gases. Under acidic conditions found in high enthalpy hydrothermal systems, CO_2_ is essentially inert and is not removed from the gas phase by interactions with hydrothermal liquids (e.g., Christenson et al. 2010). Carbon dioxide is not readily measurable using remote techniques (e.g., Schwandner et al. 2017), requiring in situ measurements. Diffuse CO_2_ degassing using accumulation chamber methods can be useful to monitor (e.g., Chiodini et al. 2005; Notsu et al. 2006; Epiard et al. 2017), although time-consuming surveys are required in often dangerous conditions.

Multiple Gas Analyzer Systems (Multi-GAS) provide a highly effective, semi-continuous, real-time and in situ method for measuring the key gas species (CO_2_, SO_2_, H_2_S; Shinohara 2005; Aiuppa et al. 2005). Gas mixing ratios (CO_2_/SO_2_ and H_2_S/SO_2_) are calculated using linear regression through concentration data points measured at a rate of 1–10 Hz. These calculations can be done in real-time. Typically, permanent Multi-GAS stations are programmed to analyze 4 times every 24 h, for a period of 30 min during each analytical session.

**Fig. 6:**
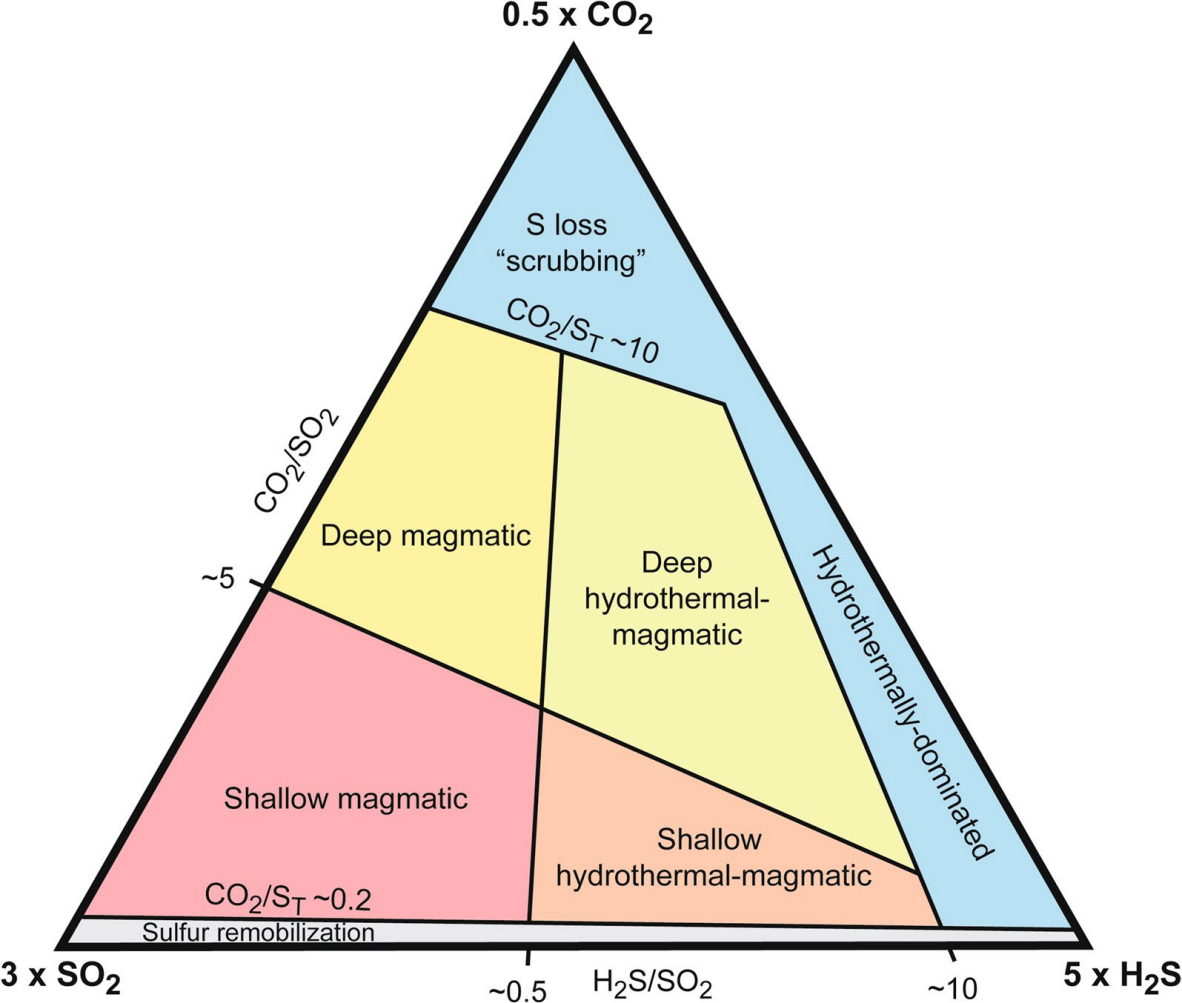
Monitoring of gas compositions can provide crucial insight into the influence of the magmatic system. Generally, as a volcanic system reactivates, the gas emissions will evolve from CO_2_-rich to SO_2_-rich. This plot shows proposed generalized fields for the characterization of hydrothermal-magmatic gas emissions, based on recent studies of Central American volcanoes (Aiuppa et al. 2014; de Moor et al. 2016a, b, 2017a). The exact boundary lines would vary with tectonic setting and other factors (Aiuppa et al. 2017), but are shown here with key gas ratio values considered appropriate for Central American volcanoes

Figure 6 shows an interpretive triangular CO_2_–SO_2_–H_2_S diagram of hydrothermal–magmatic gases, with fields useful for assessing the state of activity in volcanic systems prone to phreatic eruptions. Low temperature hydrothermal gases typical of volcanoes in a dormant state lack SO_2_ and fall along the CO_2_–H_2_S axis. As a volcano reactivates, SO_2_ becomes a significant component of gas emissions, characterized by detectable magmatic input (e.g., Vaselli et al. 2010). Gases with H_2_S/SO_2_ in the range of 0.5–10 are considered to have a significant magmatic component and are classified as hydrothermal-magmatic gases, and gases with minor H_2_S (H_2_S/SO_2_ < 0.5) are considered dominantly magmatic, without significant hydrothermal influence. Extremely sulfur-rich gases with CO_2_/SO_2_ < 0.2 are not easily explained by purely magmatic degassing processes and probably have additional sulfur contributed to the gas phase by remobilization or combustion of stored hydrothermal sulfur (e.g., Giggenbach 1987). This may be the case at Poás where CO_2_/SO_2_ ratios of < 0.1 were observed following phreatomagmatic eruptions in April–May 2017 (de Moor et al. 2017c), and also at Ruapehu prior to the September 2007 eruption when CO_2_/SO_2_ declined from 60 to 5 (Christenson et al. 2010). Within the magmatic and hydrothermal-magmatic fields, we make a distinction between deep CO_2_-rich gases and shallow SO_2_-rich gases. CO_2_ is less soluble in melt than sulfur and thus exsolves at higher pressure. As a magma rises through the crust, the first gases to reach the surface are therefore rich in CO_2_ (e.g., Giggenbach 1996). As magma continues to rise and then pond at shallow levels, the gases will become richer in SO_2_ as magma reaches lower pressure conditions allowing S to exsolve from the melt.

It is important to recognize that the classification in Fig. 6 is proposed as a generalization useful for near real-time monitoring. In dynamic hydrothermal-magmatic systems, distinguishing between gases derived from deep and shallow magmatic degassing that have also been affected by hydrothermal processes such as scrubbing (producing H_2_S at the expense of SO_2_), oxidation or remobilization of sulfur species, and mixing between gas sources, can be extremely difficult without additional information (e.g., isotopic or trace gas data; Giggenbach 1987; de Moor et al. 2016a) that is not readily available during volcanic crises.

## Concluding remarks

For type 1 phreatic systems, we propose that the likelihood of a phreatic eruption increases when the upper parts of the shallow hydrothermal system become sealed, accompanied by continued magmatic input from below in the form of high-temperature gas, supercritical fluids, and/or melt. Magma intruded at shallow levels releases large amounts of gas due to decompression (first boiling). Once emplaced in this comparatively shallow and cold environment, the magma solidifies by crystallization and further gas release (second boiling). For type 2 phreatic systems, we propose that the combined effects of shallow magmatic gas input and vaporization of the liquid-dominated hydrothermal system (typically below a crater lake) drive phreatic activity.

Both banded tremor and VLP seismic signals appear to be reliable indicators of pressurization of the shallow hydrothermal system, although the timescales of pressurization may be variable. Banded tremor pressurization timescales vary from days (e.g., Nevado del Ruiz and Etna; see Martinelli 1990; Gresta et al. 1996) to significantly longer (e.g., Karkar; see McKee et al. 1981). VLP timescales appear to be short, on the order of minutes before an eruption occurs, although they can also occur without eruption, e.g., Ontake 2007 (Nakamichi et al. 2009), which could represent a failed eruption. The additional presence of long period seismic signals is also clearly significant, indicating increased or continuing pressurization and most importantly an increased probability of eruption, as was seen at Karkar in February–March 1979 and at Ontake in September 2014.

We suggest that gas ratios may be able to play a key role in forecasting magmatically driven phreatic eruptions. At Poás, the association of decreasing CO_2_/SO_2_ and phreatic eruptive activity in April–May 2017 was a clear indication that magma was intruding to very shallow levels, in the process evaporating the hydrothermal system. At Rincón de la Vieja, another volcano in Costa Rica with a crater lake, phreatic eruptions in 2014 were associated with lowered CO_2_/SO_2_ and H_2_S/SO_2_ (de Moor et al. 2016b). Recent high-frequency Multi-GAS time series data show that individual phreatic eruptions are associated with pulses of H_2_S-poor magmatic gas (Battaglia et al. 2017).

In the opposite case, increases in CO_2_/SO_2_ and H_2_S/SO_2_ and low gas fluxes for type 1 systems could indicate a process of progressive sealing of the hydrothermal system, or scrubbing without sealing. However, if these changes are accompanied by anomalous low-frequency seismic signals indicating continued magmatic gas input to the hydrothermal system, the system is likely undergoing pressurization. In some cases, gas ratios could provide the only information that a system is sealing, e.g., Mayon volcano where no clear precursors were observed before two phreatic eruptions (Catane and Mirabueno 2001; Maeda et al. 2015b). For type 2 systems, increased CO_2_/SO_2_ and H_2_S/SO_2_ could indicate an expansion of the liquid-dominated hydrothermal system from infiltration of lake water and groundwater. Characterization of the extent and depth of hydrothermal systems at volcanoes prone to phreatic eruptions (e.g., magnetotelluric methods) and assessment of conduit conditions represent a fruitful approach for assessing the potential of volcano-hydrothermal systems to produce type 1 or type 2 phreatic eruptions, which have distinct and different implications for hazard assessment.

The process of hydrothermal sealing plays a direct role in determining the explosivity of phreatic eruptions. Understanding and recognizing this sealing process is a key direction for research and monitoring efforts. In particular, little is known about the timescales of these processes, which likely vary between different systems from decades to days, and few high-frequency interdisciplinary datasets (including both geochemical and geophysical monitoring) are available for phreatic eruptions. Chemical and physical changes may occur very slowly, as in the case of Ontake volcano, or may occur very quickly on the scale of days at more open system volcanoes such as Poás. Chemical thresholds related to temperature, pressure, pH, and redox conditions in magmatic–hydrothermal fluids clearly play fundamental roles in seal formation (e.g., Christenson et al. 2010; Rodriguez and Van Bergen 2017). Physical changes in hydrological systems and volcano plumbing systems may cause rapid precipitation of hydrothermal minerals, leading to sudden sealing. By contrast, slow chemical evolution of hydrothermal systems over time may gradually lead systems toward a state of sealing. At White Island and Ruapehu in New Zealand, condensation of magmatic vapor and precipitation of elemental sulfur and associated minerals in pore spaces of host rocks appear to exert a fundamental control on seal formation (Christenson et al. 2010, 2017). Rapid sealing is favored by (1) an interface between hot magmatic vapor and cold water causing sulfur precipitation and (2) pore space which is sufficient to accommodate and store the sulfur which is precipitated (Christenson et al. 2017).

Three key elements provide a basis for understanding magmatically driven phreatic eruptions: (1) recognizing magmatic involvement and magmatic input at an early stage of unrest, (2) identifying the timing, location, and rate of hydrothermal sealing, and (3) understanding the conditions which control vaporization of liquid water. Recognition of these three elements could help forecast and mitigate phreatic eruptions. As importantly, such recognition could be invaluable in the case of an eruption sequence which begins with phreatic eruptions and then transitions into larger and more significant magmatic eruptions.
